# A Comprehensive Literature Review of Fournier’s Gangrene in Females

**DOI:** 10.7759/cureus.38953

**Published:** 2023-05-12

**Authors:** Aisha Khalid, Sahana Devakumar, Ivan Huespe, Rahul Kashyap, Imran Chisti

**Affiliations:** 1 Research, Harvard Medical School, Boston, USA; 2 Internal Medicine, Jawaharlal Nehru Medical College, Belgaum, IND; 3 Critical Care, Hospital Italiano de Buenos Aires, Buenos Aires, ARG; 4 Research, Global Remote Research Program, Saint Paul, USA; 5 Critical Care Medicine, Mayo Clinic, Rochester, USA; 6 Critical Care Medicine, University of Miami, Coral Gables, USA

**Keywords:** diabete mellitus, female, fournier gangrene, mortality rate in sepsis, surgical debridement

## Abstract

Fournier gangrene (FG) is a rare but rapidly progressing disease with a higher mortality rate in women as compared to men. This study aims to perform a literature review about FG in females and associated mortality and morbidity. We searched databases including MEDLINE (Ovid), the National Library of Medicine (Medical Subject Headings (MeSH)), the Cochrane Database of Systematic Reviews (Wiley), as well as Embase (Ovid), Scopus, and Global Index Medicus (WHO), and reviewed literature from 2002 to 2022 and selected 22 studies that met our study’s inclusion criteria, which included 134 female patients with a mean age of 55±6 years. The perineal abscess was a more common nidus (n=41, 35%; 95%CI 23-39%) than vulvar pathology (n=29, 22%; 95%CI 15-30%). The most common initial presentation was cellulitis (n=62, 46%; 95%CI 38-55%), followed by perineal pain (n=54, 40%; 95%CI 32-50%), fever (n=47, 35%; 95%CI 27-43%), and septic shock (n=38, 28%; 95%CI 21-37%). *Escherichia coli* was the most frequently identified bacteria (n=48, 36%; 95%CI 28-46%). All patients had treatment with a mean of three (SD 2) debridement and those with negative pressure dressings received fewer debridements than those who received a conventional dressing. However, of those who had surgical intervention, 28 (20%; 95%CI 14-29%) patients underwent diversion colostomy. General surgeons performed 78% (n=104) of cases out of which 20% (n=20) were consulted by obstetrician-gynecologists, 14% (n=18) were treated by urologists, and only 8% (n=10) by plastic surgeons. The mean length of stay in the hospital was 24±11 days, and the gross mortality rate was 27 (20%; 95%CI 14-28%). In conclusion, while females have a low incidence rate of FG, they carry a higher mortality rate. Lack of cardinal signs and delayed presentation to the hospital from the onset of symptoms are some possible causes for the increased mortality rate along with the disease process being under-recognized in women. A high index of clinical suspicion is essential to avoid delay in the definitive management coupled with an early surgical consult and establishing a common general care pathway could minimize mortality and morbidity.

## Introduction and background

Fournier gangrene (FG) is a severe medical condition that involves the rapid spread of necrotizing fasciitis in the subcutaneous tissues of the perineum and external genital areas [1] and, sometimes, the abdominal wall [2]. It was initially identified in healthy young men who developed scrotal swelling without apparent cause [3]. However, the modern medical literature has now classified it as necrotizing fasciitis of the perineal, perianal, and external genitalia [4]. The disease is caused by a bacterial infection that spreads rapidly through the soft tissues of the perineal, perianal, and superficial genital regions. If left untreated, the condition can quickly cause tissue death, leading to severe complications such as sepsis, multi-organ failure, and death [5].

Although historically, the disease was limited to young men, it has been found to affect people of all ages and genders, including women and children. Early diagnosis and timely intervention are crucial for successful treatment, as untreated cases can lead to severe complications such as sepsis and multiple organ failure [6].

Various factors may increase the risk of developing FG, including diabetes mellitus, alcoholism, malnutrition, smoking, malignancies, immunosuppressive therapy, obesity, and liver or kidney failure [7]. The incidence of the disease in females is relatively low compared to males (10:1) due to better drainage of the perineal region in women through vaginal secretions [8-10]. Given the limited knowledge of FG in females, there is a need to explore the disease process in this population, assess the mortality rates, and identify the underlying risk factors and causes to improve clinical outcomes and guide effective treatment strategies. Our primary aim is to investigate FG's clinical presentation, possible etiology, and mortality in female patients via a thorough literature review. 

## Review

Methods and material

Study Inclusion Criteria and Search Strategy

Based on the guidelines of the International Prospective Register of Systematic Reviews (PROSPERO), a systematic search was conducted in several databases, including MEDLINE (Ovid), the National Library of Medicine (Medical Subject Headings (MeSH)), the Cochrane Database of Systematic Reviews (Wiley), as well as Embase (Ovid), Scopus, and Global Index Medicus (WHO) to identify relevant publications on the topic. All available and accessible data published from July 2002 to July 2022 regarding demographics, etiology, and clinical outcomes of females with FG were considered by AK and SD. All references were managed using the reference management software Paperpile (Paperpile LLC, Cambridge, Massachusetts, United States), and duplicate publications were removed before screening using the systematic review software Rayyan (Rayyan Systems Inc., Cambridge, Massachusetts, United States). Inclusion criteria were diagnosed FG on discharge or death, extractable data, and female subjects >18 years of age.

Mainly literature in English was included, and we searched MeSH terms “Female” and/or "Woman" and “Fournier’s gangrene” as both medical subject headings and crucial or free text words and included a broad range of derivations to ensure a comprehensive a search strategy as possible.

Study Selection

**Figure 1:**
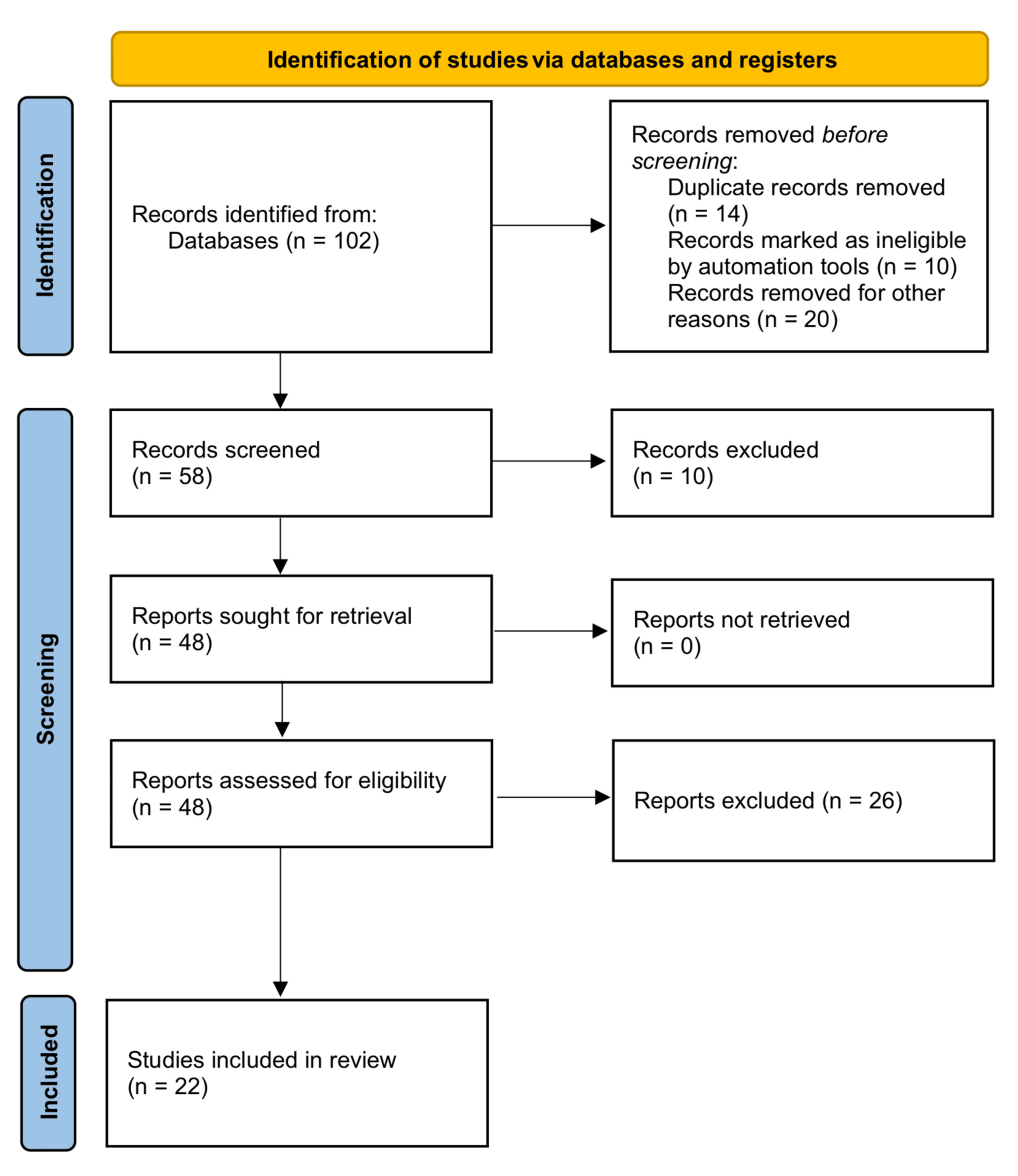
PRISMA flowchart: selected studies for Fournier’s gangrene in females PRISMA: Preferred Reporting Items for Systematic Reviews and Meta-Analyses

After a thorough screening process as explained by the Preferred Reporting Items for Systematic Reviews and Meta-Analyses (PRISMA) guidelines (Figure 1), 102 studies were identified. Fourteen were removed before the screening for being duplicated or containing non-extractable data. Finally, 22 studies were included, including one case-control study, 11 case reports, three case series, and seven retrospective cohort studies (Table 1). The most common reasons for exclusion were automated ineligible records, non-extractable desired data, an out-of-timeline search window, and incomplete data on females. 

**Table 1:** Description of included studies

Author	Year of Publication	Type of Study	Number of female participants
Ozkan et al. [1]	2014	Retrospective Cohort	5
Czymek et al. [3]	2009	Retrospective Cohort	12
Kostovski et al. [7]	2021	Case Report	1
Al Shukry and Ommen [11]	2013	Case Series	3
Unlap et al. [12]	2008	Retrospective Cohort	9
Beecroft et al. [13]	2020	Retrospective Cohort	33
Tarchouli et al. [14]	2015	Retrospective Cohort	8
Althunayyan and Karamitosos [15]	2018	Case Report	1
Basoglu et al. [16]	2007	Case Series	1
Bizet et al. [17]	2014	Case Report	1
Ferreira et al. [18]	2007	Retrospective cohort	1
Harper and Banwell [19]	2004	Case Report	1
Pehlivali and Aydin [20]	2019	Retrospective Cohort	4
Kasabwala et al. [21]	2020	Case Report	1
Nigam et al. [22]	2009	Case Report	1
Oniţa et al. [23]	2005	Case Report	1
Sobrado et al. [24]	2022	Case Report	1
Sorensen et al. [25]	2009	Case Series	39
Takano et al. [26]	2018	Case Report	1
Temiz et al. [27]	2008	Case Report	1
Vindigni et al. [28]	2016	Case Report	1
Yanar et al. [29]	2006	Retrospective Cohort	10

Data Extraction and Analysis

Each paper was assessed in two phases: screening title and abstract and then by full-text review to ensure it met the inclusion criteria. Two reviewers (AK and SD) individually did both assessments.

Predetermined study characteristics were defined for extraction and documentation. The data extracted were demographics of the female population, mean/median age with associated risk factors, common presenting symptoms, mean time to present to the hospital from the onset of symptoms, etiology, microbiology culture, and mortality and length of stay in hospital. We also looked at the surgical management plan, particularly the number of debridements required per person, the type of dressing used, and the need for colostomy. Due to the difference in the description of the microbiological cultures, we differentiated them into polymicrobial (Gram-positive, anaerobes, and fungal infections) and Gram negatives, specifically *Escherichia coli *and *Pseudomonas*.

Depending on the distribution, descriptive data was stated as mean, standard deviation or median, and interquartile range (IQR). The categorical variables were presented as proportions with a 95% confidence interval (CI). The results data collected from all selected studies were. The statistical analysis was performed with Stata 17 software (StataCorp LLC, College Station, Texas, United States).

Results

Demographic Data of the Included Studies

**Figure 2:**
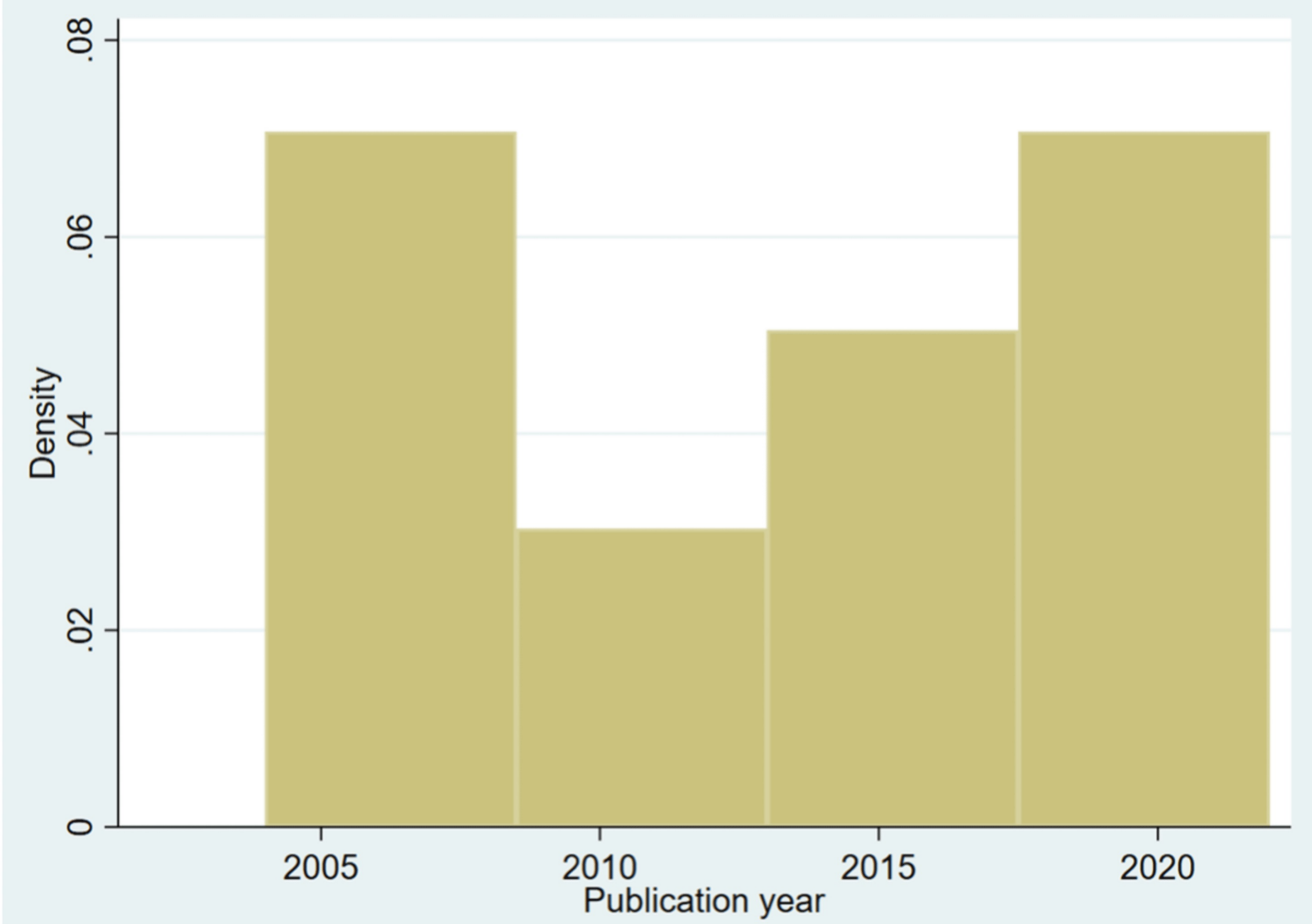
Histogram of the selected publications density from 2002 till 2022

One hundred thirty-four females with FG were included in the 22 studies evaluated. The most studied population was from the United States, 60% (n=81), and Turkey, 19.40% (n=26), as shown in Table 2. The density of the publications is shown in Figure 2. The mean age in our study was 55 (SD 6). The youngest patient was 29 years old and pregnant without any risk factors. The most common comorbidity was diabetes mellitus, with 60 females (44%; 95%CI 36-53%). Obesity was the second most common risk factor, seen in 21 females (16%; 95%CI 10-23%) (Table 3). The most common presenting symptom was cellulitis in 62 females (46%; 95%CI 38-55%), followed by perineal pain in 54 females (40%; 95%CI 32-50 %). Thirty-eight females (28%; 95%CI 21-37%) presented with severe septic shock. The mean time to present to the hospital from the onset of symptoms was seven days (SD 5). 

**Table 2:** Frequency of females per country studied

Country	Total females (n=134)
Germany	12 (8.96%)
India	1 (0.75%)
Japan	1 (0.75%)
Morocco	8 (5.97%)
North Macedonia	1 (0.75%)
Oman	1 (0.75%)
Portugal	1 (0.75%)
Romania	1 (0.75%)
Saudi	1 (0.75%)
Turkey	26 (19.4%)
United States	81 (60.45%

**Table 3:** Risk factors, comorbidities, causes, and microbiology of Fournier gangrene in females

Risk factors	Total female (n=134), n (%)	CI 95%
Diabetes	60 (44%)	36-53%
Cardiovascular disease	33 (25%)	18-32 %
Obesity	21 (16%)	10-23 %
Others	22 (16%)	10-23 %
Presenting Symptoms		
Septic shock	38 (28%)	21-37%
Perineal pain	54 (40%)	32-50%
Cellulitis	62 (46%)	38-55%
Fever	47 (35%)	27-43%
Pathogenesis		
Perianal abscess	41 (31%)	23-39%
Bartholin/vulvar abscess/necrosis	29 (22%)	15-30%
Idiopathic	03 (2.2%)	0.4-6%
Others	17 (12%)	07-19%
Microbiology		
Polymicrobial	18 (25%)	16-37%
Escherichia coli	48 (36%)	28-46%
Pseudomonas	17 (12%)	7-12%

Etiology and Microbiology

Etiologies among female patients included perianal abscess in 41 females (31%; 95%CI 23-39%) and vulvar abscess including Bartholin cyst abscess and necrosis in 29 (22%; 95%CI 15-30%). Three females (2.2.%) were deemed to have an idiopathic FG. Finally, 17 (12%) females had “other” causes including trauma, infection associated with radiotherapy in advanced cancer, and complications from prior surgeries that lead to FG. The most common microorganism was *E.coli* (n=48, 36% 95% CI 28-46%) (Table 3).

Surgical Management

Surgical management was categorized into the number of people who underwent diversion colostomy, the number of debridements, and the type of dressing used for these patients. The mean number of debridements was 3 (SD 2). Patients who underwent negative pressure dressing had a mean debridement of 2 (SD1.5). Those with conventional dressing had a mean debridement of 3.2 (SD 2.4). Most surgical procedures were performed by either general surgeons and gynecologists (n=104, 78%). Others were managed by urologists (n=18, 14%) and plastic surgeons (n=10, 8%) (Table 4)

**Table 4:** Surgical management with dressing types

Surgical Management	Total Females (n=134), n (%)	95% CI
Diversion colostomy	28 (20%)	14-29%
Vacuum dressing (negative pressure dressing)	61 (45%)	37-54%
Conventional dressing	24 (18%)	12-25 %

Morbidity and Mortality

The mean LOS was 24 days (SD 11) for all patients and the overall mortality rate was 27 (20%; 95%CI 14-28%).

Discussion

The most common sites of conspicuous gangrene in females are the labia and perineum. The etiologies among female patients, as mentioned in the studies by Czymek et al. [3] and Beecroft et al. [13] included 11 (33%) vulvar abscesses and four (12%) peri-rectal or gluteal abscesses. However, our review indicates that perineal abscess (n=41, 31%) is the most common etiology compared to vulval area (n=29, 22%) in females. The primary clinical manifestation of FG is systemic inflammatory response syndrome, sepsis, and extensive skin changes resulting from necrosis, such as red/purple patches, erythema, and swelling [30]. The most common local signs and symptoms are usually dramatic and significant pain and swelling. In our review, the most common clinical presentation was cellulitis (n=62, 42%) followed by perineal pain (n=54, 40%) and septic shock (n=38, 28%). Severe local pain and a significantly elevated white cell count on admission should alert the physician to severe infection [31].

The common cause of the delay in the diagnosis of FG is the absence of fever because the patient might be having over-the-counter fever reducers [31]. The lack of superficial signs of infection can delay diagnosing the FG. Attribution of the severe pain to some other distracting cause like surgery or pre-existing condition could be another cause of missed/ failed or delayed diagnosis [31]. In our review, only 47 (37%) female patients presented with fever. Nonsteroidal anti-inflammatory drugs (NSAIDs) may be associated with the development or progression of FG in females; however, there is some conflicting data, as per Steven et al. [31]. An FG severity index score >9 has been correlated with an increased risk of mortality [32,12]. Still, our review could not assess this scoring system in all patients due to a lack of data.

Corcoran et al. described in their study that the mean time to the hospital presentation and definitive therapy was 6.6 days with SD 4.8 in the entire cohort and was not significantly associated with mortality [32]. In our review, the mean time to present to the hospital from the onset of symptoms was seven days (SD 5), and the association with mortality is unclear. The incidence rate of FG in males in the United States is 1.6 per 100,000 [16], and even rarer in females. Despite its rarity, the disease course is associated with a high mortality rate in women of 20-50% [33], whereas in males it is 7.5%. Similar results of females having high mortality were shown by Czymeck et al. [3]. With variable data in the literature and some studies conducted showing no statistically significant difference in mortality [13,14], our study aims to add to the body of the literature to help guide the increased risk to female patients. Our review shows mortality as 27 (20%) coinciding with the predominant finding in the literature about females having higher mortality.

Diabetes mellitus is a significant risk factor in FG, i.e., 30-65% [34]. Diabetes mellitus was seen to be a significant risk factor in 44% of females, along with obesity, seen in 21% of females in our review. *E.coli *infections were the most prevalent (n=36, 48%), which is in line with prevailing literature and further substantiates the claim of a urogenital infection as the inciting cause [20,35]. Despite its importance in tailoring antibiotics, microbiological evidence is not required for aggressive and early surgical intervention [36]. Early Involvement of the surgical team is crucial to the individual’s prognosis. Numerous studies advocate for early surgical treatment, possibly accompanied by hyperbaric oxygen therapy (HBOT). Beecroft et al. found that surgical teams managing debridements in males were predominantly from urology, and female patients were overwhelmingly managed by general surgery [3]. This statement integrated well with our review, where 78% of patients were managed by general surgeons or gynecologists, and urologists or plastic surgeons saw the rest. Beecroft et al. hypothesized that the difference in surgical team management could lead to different management styles of the same condition based on gender, leading to different outcomes. Establishing general care pathways for these patients could eliminate these differences. Our review highlights the point that vacuum dressing alleviates the need for multiple debridements, which lessens hospital stay and improves outcomes [35,36] 

This review has some limitations. First, there is a lack of observations and lab values to validate the severity index. Secondly, we needed details about the ethnicity of the group involved. Finally, most of these are single-center studies, and most included studies are from the United States. Therefore, it may not represent a generalized population contributing to the observed contradictory results.

## Conclusions

Our review highlights the clinical presentation, comorbidities, etiologies, and management of FG in a cohort of 134 female patients. We found that perineal abscess was the most common etiology in females, while diabetes mellitus was the most common comorbidity. The most common presenting symptoms were cellulitis and perineal pain, while severe septic shock was present in many patients. *E. coli* was the most common microorganism isolated. Usually, superficial signs of FG may not appear until late in the course of the disease; hence, a thorough physical examination and a high degree of clinical suspicion are essential to avoid delay in the definitive management of female patients. Surgical management involves multiple debridements if the conventional dressing method is used, whereas negative pressure dressings are associated with fewer debridements. The mortality rate is high, with a mean hospital stay of 24 days and a lower incidence rate. This could indicate that some clinicians are underreported or unrecognized in this rare disease. Our review emphasizes the importance of early diagnosis and prompt surgical management, particularly in patients with co-morbidities such as diabetes mellitus and obesity. Establishing general care pathways for FG patients could eliminate management differences. Further studies are needed to investigate the effectiveness of different treatment strategies and to identify risk factors associated with mortality.
