# Correction: A Comprehensive Literature Review of Fournier’s Gangrene in Females

**DOI:** 10.7759/cureus.c273

**Published:** 2025-08-26

**Authors:** Aisha Khalid, Sahana Devakumar, Ivan Huespe, Rahul Kashyap, Imran Chisti

**Affiliations:** 1 Research, Harvard Medical School, Boston, USA; 2 Internal Medicine, Jawaharlal Nehru Medical College, Belgaum, IND; 3 Critical Care, Hospital Italiano de Buenos Aires, Buenos Aires, ARG; 4 Research, Global Remote Research Program, Saint Paul, USA; 5 Critical Care Medicine, Mayo Clinic, Rochester, USA; 6 Critical Care Medicine, University of Miami, Coral Gables, USA

This article has been corrected to fix the following typos in the PRISMA diagram:

- "Records screened (n=**62 **)" has been corrected to "Records screened (n=**58**)"
- "Records excluded (n=**14**)" has been corrected to "Records excluded (n=**10**)"

